# Long-term results after modified Steel´s triple pelvic osteotomy

**DOI:** 10.1038/s41598-025-31203-x

**Published:** 2025-12-09

**Authors:** Ondřej Schwarz, Jiří Chomiak, Pavel Dungl

**Affiliations:** 1 Department of Orthopaedics, First Faculty of Medicine, Charles University in Prague and Faculty Hospital Bulovka, Prague, Czech Republic; 2 Ortopedická klinika 1. LF UK, Fakultní Nemocnice Bulovka, Budínova 2, Prague 8, 180 81 Czech Republic

**Keywords:** Triple pelvic osteotomy, Long-term results, Harris hip score, Nonarthritic hip score, Total hip arthroplasty, Bone quality and biomechanics, Bone, Cartilage

## Abstract

Long-term results of modified Steel’s triple pelvic osteotomy were evaluated to confirm if this osteotomy is indicated in the era of other redirectional osteotomies and if it can delay total hip arthroplasty (THA). In total, 317 osteotomies were performed on 225 women and 58 men between 1985 and 2012 in one institution. Thirty-four women and three men underwent bilateral surgery. Harris hip scores (HHS) and nonarthritic hip scores (nAHS) and LCE angle, Sharp-Ulmann angle and signs of acetabular version were compared. The results were statistically evaluated using a paired t-test. In total, 55 women and 21 men were contacted. Of these, 35 women and 21 men completed the questionnaires for 68 hips. The follow up ranged from 5 to 32 years (mean 18.5 years). Of the 68 hip joints, 27 had excellent or good results according to HHS and 24 for nAHS. A total of 12 patients underwent THA. Concerning the radiological evaluation, the LCE angle increased from 12° to 34° and Sharp-Ulmann angle decreased from 49° to 36°. Signs of retroversion of the acetabulum occurred in 24 patients without of necessity of surgical treatment. Non-union (five patients) represents the most frequent complication. Steel’s triple pelvic osteotomy is still indicated as a safe and effective technique for preserving hip joints and delaying THA. The disadvantage of osteotomy is a relatively long-term recovery time and lower satisfaction compared to THA.

**Supplementary Information:**

The online version contains supplementary material available at 10.1038/s41598-025-31203-x.

## Introduction

Insufficient acetabular coverage of the femoral head may have several causes. The most frequent are acetabular dysplasia within developmental dysplasia of the hip (DDH), and secondary acetabular dysplasia within M. Perthes or due to necrosis of the femoral head (avascular necrosis within DDH, coxitis, injury). The incidence of hip dysplasia in the population is 3–4%, and approximately 0.15% for decentered hips^1–3^. Despite screening of the hips in infancy^2,4^, there are consequences in adolescents and adults in the form of residual hip dysplasia, hip subluxation or avascular necrosis of the femoral head during the treatment of DDH. These conditions represent a subsequent risk of early osteoarthritis of the hip joints and pain. Several surgical procedures have been described to reduce the consequences of these pathological conditions, improve biomechanics, and increase the load area of the hip joint^5–9^. Redirectional pelvic osteotomies help to increase the load zone of the acetabulum by changing the position of the acetabulum around the uncovered ventral and lateral part of the femoral head. Triple pelvic osteotomy represents one of these procedures. We have used Steel’s triple pelvic osteotomy^7^ since 1979, and in a modification according to Dungl since 1985^6^.

To be eligible for a triple pelvic osteotomy, the hip joint should meet certain clinical and radiological criteria. The ideal indication is a congruent hip joint on X-ray with a dysplastic, spherical acetabulum with a positive value of the LCE angle and a Sharp angle with an upper limit of 43°. From the clinical examination, at least a functional range of motion is necessary, i.e., a minimum of 60% full range of motion (FROM).

The aim of this study was to verify the effectiveness of triple pelvic osteotomy as an option to prevent or slow the development of osteoarthritis in the dysplastic hip joint, delaying the need for implantation of a total hip replacement in compliance with the indication criteria. We also wanted to find out if the acetabular retroversion, which can occur as a overcorrection of the position of the acetabulum, with femoroacetabular impingement, is a reason for surgical treatment-implatation of total endoprothesis.

## Materials and methods

The Ethics Committee of the Faculty Hospital Bulovka has reviewed and approved new research project whose results will be publishing in a journal with the title,, Long-term results after Steel´s triple pelvic osteotomy and currently indications” based on the manuscript with results of the study, and the list of all authors, Registration Number 6.6.2023/10879/EK-Z.

This study was conducted in accordance with the Declaration of Helsinki.

Informed consent was obtained from the study participants, none of whom were under 18 years of age at the time of obtaining consent to participate in the study. The study participants agreed to participate in the study by filling out a questionnaire.

**Fig. 1:**
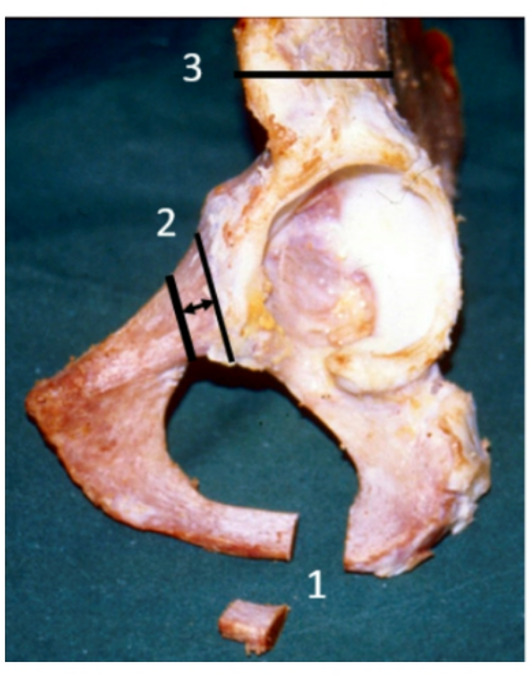
Schematic picture of the modified TPO: 1- osteotomy and resection of bone segment from the ischial bone medially from the ischial tuber; 2- osteotomy and resection of bone segment from the pubic bone medially from the eminentia iliopubica; 3 - linear osteotomy of the iliac bone proximally from the anterior inferior iliac spine.

Steel’s triple pelvic osteotomy consists of pubic, ischium, and iliac bone osteotomy with insertion of a bone graft, taken from the crista iliaca with a base width of approximately 2–2.5 cm, into the iliac osteotomy. The iliac osteotomy with the bone graft is fixed with five to seven Kirschner wires. The modification of osteotomy according to Dungl lays in strictly subperiostal resection of a 1–1.5 cm-wide segment from both the pubic and ischial bone to prevent lateralization of the hip^6^ (Fig. 1).

The surgical procedure is the same participants of all ages. The surgical technique has become routine and has enabled the surgery time to be shortened to around one hour with minimal blood loss, and without the need to start blood transfusion. No suction drainage is used. Various modified instruments were developed, including a special corkscrew, a clamp for inserting the Giglie saw through the sciatic notch, and different sizes of Hohman levers to be used according to the age and habitus of the patient.

The surgery is performed in a general anesthesia in a simple supine position. The first step is to cut the ischial bone from a separate incision in the gluteofemoral fold. We must take care not to injure the pudendal nerve, which can lead to unpleasant loss of perineal sensitiveness. Careful and tight suturing of the periosteal sleeve of the ischial bone is obligatory and prevents post-operative dislocation and the development of pseudoarthrosis.

The second part of the operation is performed through a groin incision 8 to 12 cm long. After exposure of the iliac bone, the psoas tendon is cut. The pubic bone is exposed strictly subperiostally and adjacent soft tissue structures (especially the obturator artery with its branches) are protected by a Hohman’s lever inserted medially of the eminentia iliopectinea, During osteotomy, an approximately 1 cm block is partially resected in ¾ of the pubic bone circumference.The residual cortex is osteotomized under visual control. The cortical edge is then impacted into the cancelous bone of the eminentia iliopectinea, and the periosteal sleeve is carefully sutured. The third osteotomy through the iliac bone is accomplished by subperiostally exposing the sciatic notch with special care to prevent injuries to the sciatic nerve or gluteal vessels, the Gigli saw is inserted into the sciatic notch and the ostotomy goes through the innominate line.

The bone graft, which is placed into the osteotomy of the iliac bone, is taken from the anterior part of the iliac crest. The height of the graft determines the lengthening effect of the osteotomy. The acetabular segment is transferred with a corkscrew. Five to seven Kirschner wires are used to fix both the graft and a new position to the acetabulum.

For non-cooperative children, we apply a plaster cast for six weeks. For cooperative patients, we require 10 days of bed rest and then verticalize them with sitting restrictions and non-weight-bearing for four to six weeks. After this, time partial weight-bearing is allowed. Full weight-bearing is allowed three months after surgery when all osteotomies are healed.

All the operations were performed in a single institution by a total of four experienced pediatric orthopedic surgeons.

As a cohort, we selected patients who underwent the same Steel’s triple pelvic osteotomy at our institution between 1985 and 2012. A total of 317 osteotomies were performed on 58 men and 225 women, of which 34 women and three men underwent bilateral surgery, the communication being conducted electronically. For the clinical evaluation, we used the Harris hip score (HHS) and a nonarthritic hip score (nAHS). A letter was sent to these patients requesting them to participate in the study with a request to complete the questionnaires about their condition before the osteotomy and now.

We were able to contact only 76 patients out of 283 who underwent Steele’s triple pelvic osteotomy. From the contacted patients (21 men and 55 women), 21 men and 35 women completed the questionnaires regarding the condition of their hip joints. Nine women and three men underwent bilateral surgery. The only exclusion criterion was a psychological disability that could cause non-cooperation. The mean age at the time of surgery was 21.2 years with a range of six to 39 years. The average follow-up period was 18.5 years (range of five to 32 years). Other subsequent major operations at the same time included 15 proximal femoral osteotomies and two adductor tenotomies. The K-wires were extracted from all the patients.

Anteroposterior pelvic X-ray views in a supine position were used for all surgical planning and to assess the results after osteotomy, respectively. An LCE angle below 20° and Sharp-Ulmann angle over 42° represented the main criteria for indication of surgery and both angles were used for evaluation of the results. Retroversion of the acetabulum was evaluated according to the cross-over sign^10^ and the symptom of sciatic spine, which protrudes medially. In total, we processed the X-ray documentation of 52 hip joints.

Preoperatively, the patients were divided into the following three groups based on X-ray findings on the hip joints and clinical difficulties (Table 1):

**Table 1:** Groups.

	RTG: sphericity of the head	RTG: LCE angle	RTG: AVN O-B	Clinical
Group 1	+	0–15°	type II	no pain
				full range of movement
Group 2	-	negative	type III	pain, limp
				limited motion
Group 3	-	-	type I + II	pain, limp
				limited motion

**Fig. 2:**
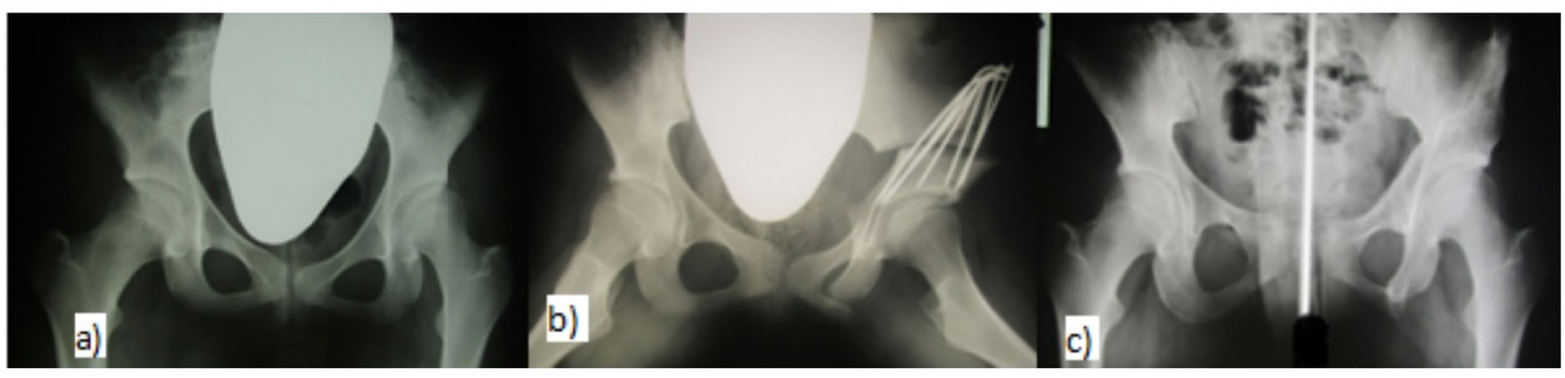
Patient with an ideal indication-asymptomatic hip dysplasia on the left **(a)** before osteotomy at 15 years, **(b)** after surgery, **(c)** excellent result after 16 years without signs of osteoarthritis.

Group 1 - ideal indications. This group includes the patients with a spherical hip joint and residual hip dysplasia, with no or minimal degenerative changes (Tönnis grade I of arthrosis), with a full range of motion and with no pain or symptomatic pain after exercise (Fig. 2A-C). This group also includes patients with sequelae of type II avascular necrosis (AVN) according to Bucholz and Ogden (B-O)^11^. The sphericity of the femoral head is altered only minimally, but the large craniolateral part of the head protrudes outside the acetabulum. The LCE angle is in the range of 0 to 15°. A total of 26 patients were included in this group, who only had radiological findings without clinical symptoms, seven of whom underwent bilateral surgery.

**Fig. 3:**
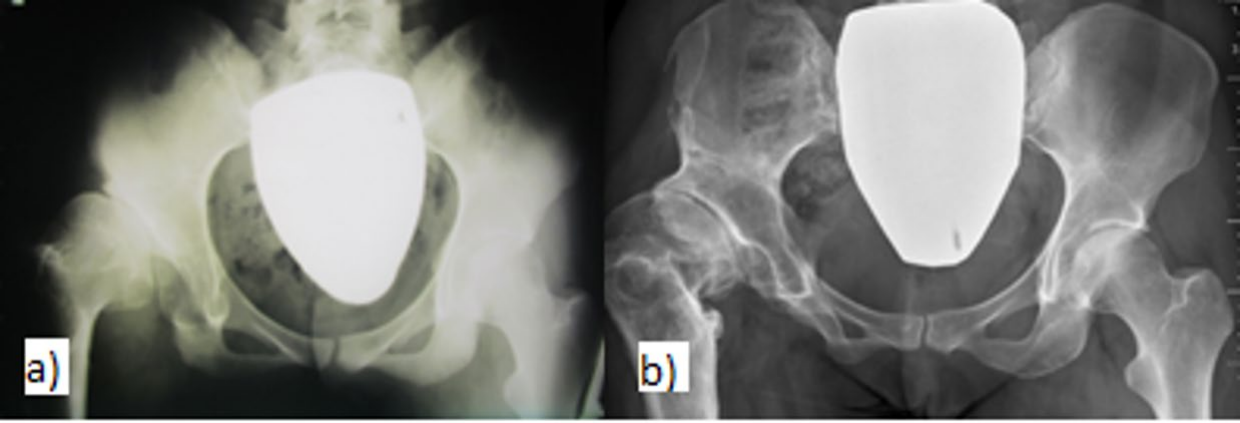
Patient with extended indication-type IV AVN deformed head according to O-B after conservative DDH treatment on the right **(a)** before surgery at the age of 18, **(b)** after 22 years with the development of arthritic changes.

Group 2 - extended indications. This group includes patients with a symptomatic hip joint with an incipient deformity, limited movement, and decentration. The LCE angle was 0° or negative. The femoral head may be subluxated but not dislocated (Fig. 3A-B). Patients with post-dysplastic type III AVN according to the B-O classification were also included in this group. This consists of a deformed head of the femur, shortening of the femoral neck, and overgrowth of the great trochanter. This group contained 22 patients, five of whom underwent bilateral surgery.

Group 3 – necrotic head. This group includes patients after necrosis of a larger or smaller part of the femoral head. This group comprised eight patients, of whom four had Perthes disease, two had coxitis, and two had an injury. For the patients with Perthes disease, the X-ray findings were classified according to Stulberg^12^ (group I-1 patient, group III-2 patients, group IV-1 patient). The X-ray findings for the patients after coxitis corresponded in one patient to type II-A and in the other to II-B according to Hunka^13^. In two patients with post-traumatic necrosis of the femoral head, the findings corresponded to grade I and II according to B-O.

### Statistical evaluation

The preoperative and postoperative values in the same patients were compared using a paired t-test. We had a sufficient number of evaluated X-ray images (52 hips, 93%) to statistically process the values of the LCE angle and Sharp-Ulmann angle. Statistical evaluation of the change in HHS and nAHS from the small number of questionnaires completed for both the preoperative and postoperative condition compared to the number of questionnaires completed only for the postoperative condition was more problematic. Missing data cannot be fully replaced. Assuming the similarity of groups of patients with and without preoperative HHS, methods can be used that, based on the relationship (correlation) between preoperative HHS and at the end of the follow-up for complete records, are able to estimate the preoperative HHS value in the remaining patients and then the comparison may be performed based on the supplemented data. The method used is a repeated measurement model for unbalanced data with a structured covariance matrix. For nAHS, the statistical evaluation was even more limited because even fewer patients completed the preoperative and postoperative questionnaires. The ability of the statistical methods to demonstrate the difference decreases with the number of observations.

## Results

### Clinical results

We determined that in 34 patients, the greatest issue was pain during exercise and limping when the limb was short-circuited because of hip dysplasia or its treatment. Eleven patients limped without pain. Only five patients had no clinical symptoms with X-ray findings of clinically silent hip dysplasia before surgery.

A total of 56 patients with disability of 68 hip joints completed the questionnaires regarding the current condition or the condition prior to total hip replacement. Only 12 patients completed the preoperative questionnaire for 13 hip joints.

The mean HHS was 58 points with a preoperative range of 38 to 94 points. At the end of the follow-up, or before THA, the mean HHS was 67 points with a range of 15 to 99 points. There was no statistically significant change (*p* > 0.4) with a standard deviation of 33.2. When evaluating the whole cohort using the statistical method for unbalanced data, a statistically significant change in HHS with *p* < 0.05 was obtained. The mean value for nAHS for the six patients who completed the preoperative questionnaire was 53 points with a range of 30 to 70 points. The mean nAHS at the end of the follow-up or before THA was 75 points with a range of 15–100 points. When we compared the data for these six hip joints, the difference was statistically significant (*p* < 0.05). When we applied the method for unbalanced data, there was a very small value of *p* < 0.001, which indicates a statistically highly significant change. We found excellent and good results with a score above 80 in 24 hip joints according to HHS and in 27 hip joints for nAHS. Satisfactory and poor results were recorded in 40 hip joints with an HHS below 80 points and in 37 hip joints for nAHS, including 12 THA with a time to implantation of 14 years (4–31). The clinical results are summarized in Table 2.

**Table 2:** Clinical results.

	Preoperative	Postoperative	Improvement
**Overall**			
Pain	47	38	9
Limping	51	37	14
HHS	58 (38–94)	67 (15–99)	9
nAHS	53 (30–70)	75 (15–100)	22
** 1 st group**			
Pain	26	18	8
Limping	28	15	13
HHS		69 (15–98)	
nAHS		78 (25–100)	
**2nd group**			
Pain	24	13	11
Limping	25	14	11
HHS		62 (29–94)	
nAHS		71 (40–95)	
**3rd group**			
Pain	7	7	0
Limping	0	1	−1
HHS		74 (63–95)	
nAHS		81 (71–95)	

In the first group of ideal indications, the results were excellent and good according to HHS in 13 hip joints out of 33 and according to nAHS in 12 hip joints out of 33. Satisfactory and poor results were found in 20 and 21 hip joints, respectively. The mean HHS in this group was 69 points and in the case of nAHS it was 78 points. In this group, a clinical condition without pain and without limping was achieved in 14 hip joints (42%). We performed THA on three patients, with a time to implantation of 14 years (14–16). The mean follow-up was 17.3 years (5–30).

In the second group of extended indications, we recorded a mean HHS of 62 points and nAHS of 71 points in 22 patients and 27 hip joints, respectively. Nine out of 22 patients reached more than 80 points in HHS or nAHS. In 13 patients (48%), we reached a condition where the joints could be fully burdened without pain and without limping. In this group, we performed THA on nine patients, with a time to implantation of 14 years (4–31). The mean follow-up period was 18.9 years (7–26).

In the third group, the mean HHS was 74 points and nAHS was 81 points, with a very good and good result in three patients. Only one patient (33%) in this group was without pain and without limping. We did not have to perform THA on any of the patients. The follow-up period was 19.3 years (8–27).

If we divided the set into a group of pediatric and adult patients, we would find slightly different clinical results for both groups (Table 3).

**Table 3:** Clinical results for children and adults.

	Number of hips	Age	follow-up	HHS	nAHS	THA
Children	33	13,6	21,6	75,5	76	5x
Adults	35	27,9	16,6	67	69	7x

In the pediatric patients, the mean HHS score was 75.5 points (15–99) and the nAHS score was 76 points (25–100). Excellent and very good results were observed in 12 patients according to HHS and 11 patients according to nAHS, respectively. Satisfactory and poor results were observed in 12 patients according to both HHS and nAHS. The mean follow-up period was 21.6 years (7–32). In the group of pediatric patients, we implanted THA a total of five times. The age at the time of osteotomy was 13.6 years (6–17). The time until THA implantation was 20 years (13–32).

In the adult patients, the results are somewhat worse with shorter follow-up. The mean HHS and nAHS scores were 67 (29–96) and 69 (15–99) points, respectively. Excellent and very good results were found in 12 patients according to HHS and in 14 patients according to nAHS. In 21 patients, the results were satisfactory and poor according to HHS and in 23 patients according to nAHS. The mean follow-up was 16.6 years (5–27). In the group of adult patients, we implanted THA a total of seven times. The age at the time of osteotomy was 27,9 years (20–39) and the time until THA implantation was 12.6 years (5–21). All data are available in Additional file 1.

### Radiological results

From the available X-rays, the LCE angle improved from an average preoperative value of 12° (range from − 24° to 42°) to an average postoperative value of 34° (range from − 3° to 62°) (Table 3). This resulted in a statistically significant increase in the LCE value with *p* < 0.001 and a standard deviation of 12.5°. The Sharp-Ulmann angle decreased from 49° (range 38°- 63°) to 36° (range 22–59°) (Table 4). There was a statistically significant decrease in the Sharp-Ulmann angle with *p* < 0.001 and a standard deviation of 6.2°. In the first group, the LCE angle increased on average by 21° (range 18° −56°) from a preoperative 15° to 36° and the Sharp-Ulmann angle decreased on average by 14° (range 22°−50°) from 48° to 34°. In the second group, the LCE angle increased from an average of −2° (range − 24°−9°) to 26° (range − 3°−51°). The Sharp-Ulmann angle decreased from 53° (range 41° −63°) to 39° (range 23°−59°). In all of the patients in this group, the X-ray incipient deformity of the head was evident prior to surgery. In the third group of patients, the LCE angle increased from 29° (range 19° −43°) to 47° (range 42° −53°) and the Sharp-Ulmann angle decreased from 45° (range 42°−47°) to 32° (range 22°−37°). In this group, a smaller or larger deformity of the head was present in all cases. Patients who underwent THA of the hip joint showed signs of advanced osteoarthritis on their X-rays. All data are available in Additional file 2.

**Table 4:** Radiological results.

	Preoperative	Postoperative	Improvement
**Overall**			
CE angle	12° (−24°−42°)	34° (−3°−62°)	22°
Sharp-Ulmann angle	49° (38°−63°)	36° (22°−59°)	13°
** 1 st group**			
CE angle	15° (11°−19°)	36° (18°−56°)	21°
Sharp-Ulmann angle	48° (39°−52°)	34° (22°−50°)	14°
**2nd group**			
CE angle	−2° **(**−24°−9°**)**	26° (−3°−51°)	28°
Sharp-Ulmann angle	53° (41°−63°)	39° (23°−59°)	14°
**3rd group**			
CE angle	29° (19°−43°)	47° (42°−53°)	18°
Sharp-Ulmann angle	45° (42°−47°)	32° (22°−37°)	13°

The cross-over sign was present in 14 hip joints preoperatively and increased to 38 hip joints postoperatively. Newly occurring retroversion did not lead to another operation or even THA.

### Complications

None of the patients suffered any serious infection or severe neurovascular injury, and none of the patients complained of hypesthesia or anaesthesia in the innervation area of the cutaneous femoris lateralis nerve. Non-unions occurred in five cases. Separate non-unions occured after osteotomy of the ischial bone (two patients) and were asymptomatic. Non-unions required surgical treatment in three patients. The first patient developed a non-union of the iliac bone; in the second patient, not three osteotomies healed, the hip joints were symptomatic and required early revision with cancellous bone application and osteosynthesis. The third patient was indicated up to 15 years after surgery for osteosynthesis and a cancellous bone graft for the non-unions of the pubic and iliac bones. None of these complications had any influence on the long-term results.

## Discussion

The aim of this work was to defend the indication of this operation even in the era of modern redirectional osteotomies and total hip joint replacement. According to our findings, triple pelvic osteotomy is a safe method of treatment of residual hip dysplasia and subluxation with a minimum of serious complications, providing relatively good results in the long term. Due to the reported average survival rate of THA of 20–25 years^14^, at least one re-implantation or revision of THA may be avoided using triple pelvic osteotomy.

Disadvantages of this osteotomy include the fact that it requires a longer rest period due to its instability and relatively often leads to acetabular retroversion^15,16^. These disadvantages can be justified by the fact that at the time of the initiation of the follow-up in the 1980 s, periacetabular osteotomy and knowledge of the signs of acetabular retroversion were not widespread. Nevertheless, none of the patients in our group with acetabular retroversion underwent surgery for clinical issues in terms of femoroacetabular impingement. Compared to Castaneda^17^, we did not find clinically significant acetabular retroversion, although it was present in 27% of patients before surgery and in 73% of patients after surgery.

The decision to provide surgical treatment of residual hip dysplasia in adolescents and young adults with only post-exercise pain is still a dilemma. The incidence of secondary osteoarthritis because of congenital hip dysplasia is reported to be in the range of 25–58%^15,18–20^. Coopermann et al.^21^ stated that subluxation in the hip joint leads to early osteoarthritis the same as in the case of asymptomatic hip dysplasia without signs of subluxation, but over a longer period. Redirectional pelvic osteotomy was developed with the aim of delaying the onset of osteoarthritis, which inevitably occurs in dysplastic hip joints, or a significant slowing of the development of arthritic changes. From this point of view, we may consider triple osteotomy as a preventive method.

We fully agree with many other authors^5,9,19,22,23^ that changing the position of the acetabular segment leads to improved biomechanics of the hip joint but does not allow the restoration of normal hip joint anatomy. Some authors report good to excellent results in a 10-year follow-up period. Guille et al.^18^ observed radiological improvement in 10 of 11 hip joints and a clinical improvement in eight of 11 patients with a mean follow-up of 12 years in a small cohort. Hailer et al.^24^ examined 51 patients (61 hip joints) treated with a triple Tönnis osteotomy with a mean follow-up of six years. An excellent or good result was observed in 68% of cases. Van Hellemondt et al.^25^ describe excellent and good results in 56% of cases over a 15-year follow-up period, but in their group, the patients only had a spherical femoral head without signs of osteoarthritis.

We recorded excellent and good results in 42% of cases and unsatisfactory and poor results in 58% of cases. This may be due to several factors. One is the length of follow-up, which averaged 18.5 years, compared to other studies. Similarly, in our group there were patients not only with asymptomatic hip dysplasia, where the results were significantly better.

Clinical results with respect to the patient’s age at the time of surgery are better in the pediatric patients. In the pediatric patients, 50% had very good and good results, while in the adults it was only 33%. This is probably due to the ability of the acetabulum and femoral head to remodel when the load changes after the osteotomy. Pediatric patients also adapt better to a worse health condition and in adulthood consider it as normal what an adult patient would consider bad.

The clinical results according to the division into groups of ideal, extended, and borderline indications, are different than expected. The worst results are in the group of ideal indications, where only 12 (36%) of 33 patients had excellent or very good long-term results. This is probably due to the fact that previously healthy patients with only X-ray signs of dysplasia were operated on, and any such major operation can obviously leave damaged soft tissues and muscles. Similarly, a change in the load on individual parts of the hip joint can cause faster degenerative changes. On the other hand, we know that hip dysplasia is a pre-arthrotic condition that irreversibly leads to the development of osteoarthritis and subsequently to the implantation of a THA.

Compared to the Bern periacetabular osteotomy, which is probably the most widely used conservative procedure for residual hip dysplasia today, we have a lower THA implantation rate as Steppacher et al.^26^. (20% vs. 40%). Otherwise, our clinical results (limping, functional scores) are very similar, as are the radiological results (improved head coverage), with the exception of an increased number of acetabular retroversions. However, the clinical results at the end of the follow-up are satisfactory or poor in 40% of patients, and it may be assumed that we will have to implant a THA in the near future.

As a great advantage of the Bern periacetabular osteotomy, we see greater variability in the reorientation of the acetabulum and the earlier possibility of loading the operated side^27^. The disadvantage is a more difficult surgical technique and a longer learning curve. Likewise, there is greater blood loss during this operation with the frequent need for blood transfusions. From the radiological results, the biggest difference is in the postoperative incidence of acetabular retroversion, which is significantly higher after triple pelvic osteotomy.

The limitation of our study is that it is a single institution study, and the relatively small sample of patients due to their unwillingness to cooperate or the inability to contact them. The reasons for this include the relatively large difference in time between the performed operations and the start of the study, changes in address, a change of name, or death.

Statistical evaluation of the clinical results is limited by the very small amount of data on the clinical condition of the hip joints before surgery due to the relatively long time since surgery or the reluctance to complete the same questionnaires twice. Therefore, we determined the issues that the patients had before surgery not only from the questionnaires but also from their medical records.

The strength of our study is that it evaluates the long-term results of triple pelvic osteotomies using the same technique of one institution.

The number of triple pelvic osteotomies is decreasing every year, at least at our hospital. We believe that this is due to several reasons. Most importantly, hip dysplasia screening has become widespread with the introduction of early ultrasound diagnosis and the possibility of initiating early and effective conservative therapy^2^. Similarly, surgical procedures around the hip joint are approached at a younger age in order to utilize the ability to remodel the hip joint socket^8^. At the same time, other surgical methods are being developed that do not require a complete pelvic osteotomy with a consequent change in the position of the acetabulum (Bernese periacetabular osteotomy)^7,26,28^. Currently, we still have triple pelvic osteotomy on the list of procedures that we can use in the treatment of residual hip dysplasia as well as periacetabular osteotomy or endoprostheses. Indications of triple pelvic osteotomy nowadays are relatively narrow. It is acetabular dysplasia and subluxation after DDH or Perthes disease. Ideally the X-ray image should not show significant dysplasia with LCE around 0° without signs of arthrosis^29,30^. A triple osteotomy can correct even larger head cover defects, but at the cost of a more pronounced postoperative limitation of mobility. Further indication is represented by mild acetabular dysplasia in skeletally mature patients where acetabular correction may be effectively achieved without acetabular retroversion. A triple osteotomy is also indicated in the case of triradiate cartilage still present, when it is not possible to perform a periacetabular osteotomy. The clinical finding is dominated by pain after exercise, for which the patient sought medical help. The patient must agree with the operation and its possible risks after a proper explanation of the issue, including the offer of other operational services. The advantages of TPO are that it is a relatively simple operative technique, short surgical time, low blood loss, and a low complication rate.

It is always necessary to have several options available to address a given condition and, after considering all the circumstances, decide on the most appropriate option. Even today, there are patients for whom a triple osteotomy is a suitable operation. As already mentioned, it is a less demanding operation, both for the patient and the surgeon, than the Bernese periacetabular osteotomy, which is probably the most widespread in adolescents and adult patients today^31,32^.

## Conclusion

Triple pelvic osteotomy in Dungl’s modification provides good long-term results and delays the need of a total hip arthroplasty, when correctly indicated in groups of patients 1 and 2. This operation is not a problem for a more experienced surgeon and he or she is able to learn it within a few operations and then perform it in about one hour. Likewise, it does not pose any major risks of injury to large vessels and nerves when operated carefully. The advantages of this osteotomy are the relatively short operating time and therefore small blood loss and no need for special instruments. The disadvantages are a relatively long recovery time, possible retroversion of the acetabulum with subsequent femoroacetabular impingement, and lower satisfaction compared to THA.

## Supplementary Information

Below is the link to the electronic supplementary material.

Supplementary Material 1

Supplementary Material 2

## Data Availability

The datasets used and/or analyzed during the current study are available from the corresponding author on reasonable request.

## Code availability

Statistical analysis one-sided Student’s t-test was performed by Stata statistical software, release 9.2, Stata Corp. LP, College Station, TX.
